# Breaking away from labels: The promise of self-supervised machine learning in intelligent health

**DOI:** 10.1016/j.patter.2021.100410

**Published:** 2022-02-11

**Authors:** Dimitris Spathis, Ignacio Perez-Pozuelo, Laia Marques-Fernandez, Cecilia Mascolo

**Affiliations:** 1 Department of Computer Science and Technology, University of Cambridge, CB3 0FD Cambridge, UK; 2 MRC Epidemiology Unit, School of Clinical Medicine, University of Cambridge, CB2 0SL Cambridge, UK; 3 Addenbrooke's Hospital, Cambridge University Hospitals NHS Foundation Trust, CB2 0QQ Cambridge, UK

**Keywords:** machine learning, health signals, transfer learning, biomedical informatics

## Abstract

Medicine is undergoing an unprecedented digital transformation, as massive amounts of health data are being produced, gathered, and curated, ranging from in-hospital (e.g., intensive care unit [ICU]) to person-generated data (wearables). Annotating all these data for training purposes in order to feed to deep learning models for pattern recognition is impractical. Here, we discuss some exciting recent results of self-supervised learning (SSL) applications to high-resolution health signals. These examples leverage unlabeled data to learn meaningful representations that can generalize to situations where the ground truth is inadequate or simply infeasible to collect due to the high burden or associated costs. The most prominent bottleneck of deep learning today is access to labeled, carefully curated datasets, and self-supervision on health signals opens up new possibilities to eliminate data silos through general-purpose models that can transfer to low-resource environments and tasks.

## The bigger picture

Machine learning (ML) touches every area of science, and medicine especially is well poised to benefit the most. Hospital and nonhospital settings generate unprecedented amounts of data that if used correctly can unlock advances in new diagnostics and contribute to preventive medicine. The established paradigm of ML (supervised) requires the collection of input data (such as vitals or imaging) coupled with annotations from experts (such as indications of arrhythmia). New self-supervised models promise to do without annotations by using clever transformations of the input data only and achieve remarkable performance in an array of clinical tasks. This perspective gives a brief overview of the fundamental methodologies that enable these advances and discusses further challenges and opportunities.

## Teaser

In recent years, there has been increasing interest in applying artificial intelligence (AI) to biomedical signals, with rapid progress in various applications using wearable sensors or vital signs. However, it remains challenging to develop robust models because large datasets are often scarce due to the time-consuming effort required for annotation. New models leverage larger datasets because they do not require annotations and open up new possibilities to eliminate data silos.

## Introduction

### Underutilized medical data and the label gap

Medical data have the power to transform lives. Advances in the ways in which we collect, process, interpret, and use these data can be used to save lives and transform our society. Although the overwhelming majority of current medical research now focuses on clinical data (labs, imaging, vitals, etc.), the average person visits a doctor only around five times a year. Further, recent advances in wearable sensing and mobile computing, alongside their widespread and growing adoption, have created new pathways for the collection of health and well-being data outside of the laboratory and hospital settings, in a longitudinal fashion. These devices can be used to “fill the gaps” that are often found in traditional clinical data, opening up new research and commercial directions for large-scale lifestyle monitoring and providing sources of truth in nondisease scenarios. For example, millions of people worldwide use such devices to track their physical activity and sleep,^1^ with increasingly more sophisticated predictive capabilities and a wider range of sensors used to monitor these human behaviors and activities.

Concurrent to this self-monitoring revolution, seemingly disparate forces such as mature open-source scientific software libraries, easier data crowdsourcing and labeling, and the repurposing of specialized hardware (graphics cards) have enabled dramatic improvements in predictive modeling. Many machine learning (ML) tasks have achieved impressive performance, ranging from object recognition in images to outperforming experts in breast cancer screening. The common denominator in all these cases has been the curation of high-quality large datasets that allow models to exploit latent patterns and subsequently generalize in real-world scenarios. However, especially in medicine, where erroneous predictions can have grave consequences, the roll-out and adoption of such systems have been met with resistance, mostly citing algorithm interpretability reasons.

Similar to how social networks learn our online behaviors, wearable and mobile devices monitor our activities in the real world. By tracking our sleep, steps, and eating and working habits, they create a holistic understanding of the most important components of our everyday health, until now only possible through subjective surveys. Although we recognize the value of such datasets, advances in ML for health and mobile sensing have not kept up with other areas. For example, over the last decade devices such as Fitbit or the iPhone have been collecting multi-modal sensor data at an unprecedented temporal resolution. However, effectively leveraging these datasets has presented many challenges, leading to these data being frequently overlooked for scientific and medical research.^2^ Central to this problem is obtaining quality annotations and ground truth, which can be costly, burdensome, and at times, even impossible, given the granularity of these data. In this article, we discuss the potential of self-supervised methods toward bridging the label gap in biomedical data.

## Breaking away from labels

### Supervised learning: Reaching its limits

Deep supervised learning requires a decent amount of labels and samples in order to achieve good performance. (Arguing about the optimal dataset/label size is definitely a very empirical problem at this moment and that is why we do not quote exact numbers here, since it depends on the complexity of the given problem and the model. There is evidence that accuracy plateaus faster with supervised models compared to self-supervised ones, and the gains are mostly in the low-data regime.^3^) These manual labels—in the best scenario—are easier to obtain through crowdsourcing (cf. Imagenet), but in some cases, it is virtually impossible. For example, annotating wearable sensor timeseries for human activity recognition tasks *a posteriori* is not feasible without a video recording. On the other hand, given the fact that the amount of unlabeled data (e.g., all the images on the internet or the entire Fitbit user base) is considerably more heterogeneous and representative than some limited datasets, ongoing research and interest in this area have grown significantly. However, unsupervised learning is hard and, until recently, was less efficient than supervised learning. The first promising unsupervised studies in the area of health signals employed the successful paradigm of word2vec and reported results on a par with supervised models.^4^ However, the static vectors produced with these methods have limitations against context-specific representations.^5^

### Self-supervised learning: The quest for the best data representation

**Figure 1:**
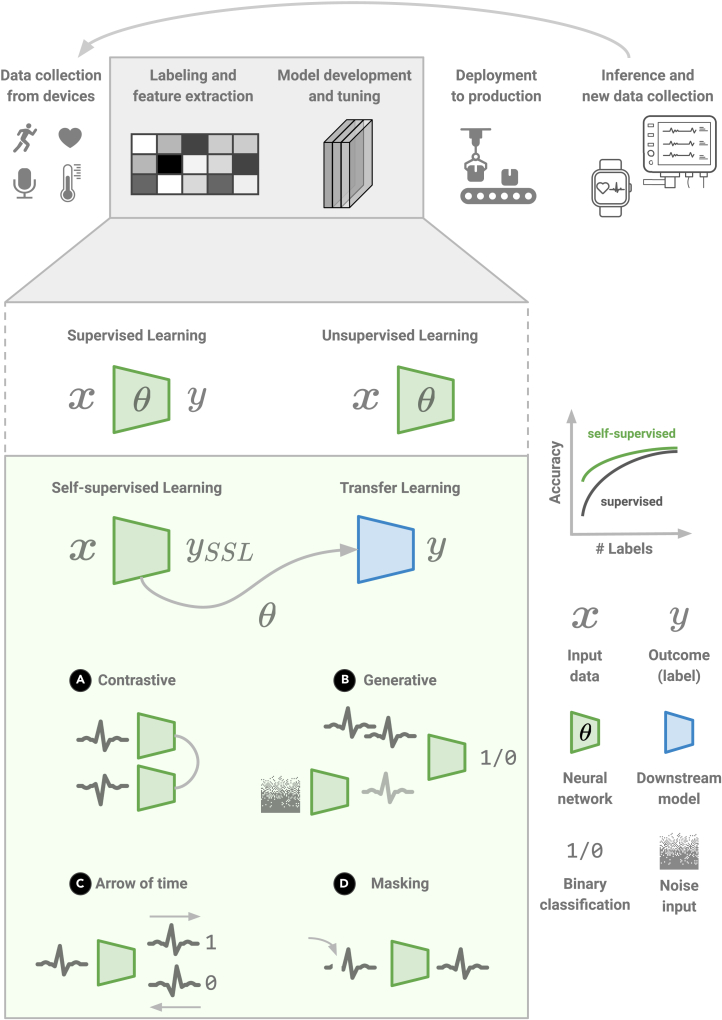
Self-supervised learning for health signals Here, we illustrate the case of ECG signals and the prominent methods, which leverage unlabeled data with self-supervised learning. (A) Contrastive training maximizes the agreement between the original and the distorted view (flipped, rotated, or other augmentations). (B) Generative models such as GANs involve two networks that contest with each other in a game to generate more realistic data. (C) Time-aware models try to guess whether the signal follows the arrow of time. (D) Masked models hide part of the signal and challenge the model to predict the original one. The representations learned from these methods can be reused on downstream transfer learning tasks with linear models (blue box). Self-supervision is more label efficient in low-data regimes (top right graph).^3^

A simple yet exciting emerging idea is to obtain labels “for free” from the input data (**x**) through various transformations and, then, use conventional supervised objectives to predict them ($y_{SSL}$). The representations obtained this way would be meaningful for downstream tasks with limited labeled data and linear classifiers (see Figure 1). This has been coined self-supervised (or predictive) learning (SSL) due to learning the supervision directly from the data. (The terminology surrounding unsupervised and SSL is a bit blurry. Unsupervised learning is used for a wide range of models, ranging from autoencoders^6^ and Boltzmann machines^7^ to principal component and cluster analysis. SSL can be seen as a subset of unsupervised learning, where supervisory signals are learned directly from the data.^8^ However, the two terms are sometimes used interchangeably.) Even before this term was coined, researchers used to handcraft pretext tasks, which exploited unlabeled data. The most common tasks involved predicting distorted versions of the spatial characteristics of image data by means of rescaling, rotating, patching, shuffling, colorizing, and inpainting missing parts.

However, one could argue that devising these increasingly complex pre-training tasks resembles traditional feature engineering that neural networks promised to automate. Therefore, more generic recent methods have switched their focus from inventing single data transformations to comparing such views in the latent space and, therefore, offering elegant methods of implicit clustering between pseudo-positive and negative samples. Notably, SimCLR^9^ achieved—for the first time—performance on a par with supervised models, by proposing a two-network training method for visual representations, which maximizes agreement between differently transformed views of the same sample via a contrastive cosine similarity loss in the latent space. More recently, BYOL claimed better results even without the negative pairs in its training objective through a similar two-network approach.^10^ This sounds surprisingly similar to another family of models: generative adversarial networks (GANs), where the objective draws from game-theoretic principles, and two networks contest with each other in a game to generate more realistic data (see Figure 1). A useful taxonomy is introduced in,^11^ where SSL models are grouped into three categories: generative (e.g., autoencoders), contrastive (e.g., SimCLR), and generative-contrastive (e.g., GANs or adversarial autoencoders). The main difference across these categories is the objective, ranging from reconstruction and contrastive losses to distributional divergences. We expect to see more overlap between generative, adversarial, and contrastive training in the future.^12^

These methods have shown promise that can indeed generalize to other data beyond images; however, data augmentations or objectives might need to be adapted when moving to a different type of input data.^13^^,^^14^ Furthermore, approaches such as the arrow of time^15^ exploit the temporal—rather than spatial—information of the data, by either artificially reverting the input sequences so as to distinguish between the correct and the reverse order (see Figure 1), or just predicting the future. We believe that models that anticipate and forecast the future are more robust and generalizable (for example, across hospitals).^16^ This seems to be particularly effective in language tasks as well, where models such as GPT-3^5^ outperform every other method by just slicing the data in such a way so as to predict the next word.

### Transfer learning: The art of fine-tuning

Transfer learning is the natural application of SSL. The term transfer describes a set of methods toward preserving and reusing previously acquired information, applied possibly to a slightly different domain. This stored information can further accelerate the training of a downstream task with usually limited training data. Modern transfer learning uses pre-trained networks as fixed feature extractors in a) linear downstream models, where a logistic regression classifier is trained to classify a new dataset based on the self-supervised embeddings, or b) further fine-tuning the model in a downstream task. This has shown remarkable results in vision and language domains, where the learned embeddings can be directly applied to smaller datasets.^5^^,^^9^

## Self-supervision for health signals

We will now present some recent results of self-supervised models applied to biomedical signal data (for a comprehensive view of this topic, the reader may be interested in Chowdhury and colleagues' review on SSL on medicine).^17^

### Learning generalized physiological representations

While everyone can download off-the-shelf pre-trained models to further customize vision or language tasks, this is not the case for health signals. To this end, we recently developed Step2Heart,^18^ a self-supervised model that exploits the multi-modal data of modern wearables to learn meaningful representations, which generalize to several outcomes with transfer learning. The model maps activity data to future heart rate (HR) responses (implicitly applying the arrow of time principle) and can be used as a feature extractor for wearable data. For pre-training, we used a joint quantile loss function that accounts for the long tails of HR data, while downstream, we aggregated the window-level features to user-level ones and showcased the value captured by the learned embeddings through strong performance at inferring physiologically meaningful variables, ranging from anthropometrics to fitness, outperforming unimodal autoencoders and common biomarkers. For instance, the embeddings achieved an area under curve (AUC) of 0.68 for cardio-fitness prediction and an AUC of 0.80 for physical activity energy expenditure. Obtaining these outcomes in large populations can be valuable for downstream health-related inferences that would normally be costly and burdensome (for example, a fitness test requires expensive laboratory treadmill equipment and respiration instruments). Additionally, low fitness is a stronger predictor of mortality than diabetes, hypertension, and smoking.^19^ Overall, this is one of the first multi-modal self-supervised models for behavioral and physiological data, with implications for large-scale health and lifestyle monitoring (open source code and pre-trained models are available on https://github.com/sdimi/Step2heart).

### Improving human activity recognition

A staple task in mobile health is that of human activity recognition (distinguishing between walking, sitting, running, etc., through activity sensors). This task is fundamental for the development of higher-level health monitoring applications. With SelfHAR,^20^ we recently showed that large unlabeled mobile accelerometer datasets can be leveraged to complement small, labeled ones. Our approach combines teacher-student self-training, which distills the knowledge of unlabeled and labeled datasets, while allowing for data augmentation, and multi-task self-supervision, which learns robust signal-level representations by predicting distorted versions of the input. SelfHAR achieved up to a 12% increase in performance (F1 score) using the same number of model parameters, by using up to 10 times fewer labeled data compared to supervised approaches. This work showed how to effectively distill, filter, and use unlabeled data orders of magnitude larger than supervised datasets. In a subsequent study, we studied the impact of the combinations of timeseries transformations in SimCLR models for activity recognition.^13^

### Subject-aware biosignal representations

Commonly, datasets with a small number of subjects, such as electroencephalograms (EEG), manifest high intersubject variability. Therefore, Cheng et al. proposed a self-supervised model with an adversarial subject identifier to minimize subject-specific content.^12^ They developed domain-inspired augmentations such as frequency-based perturbations to augment the signal, because the power in certain EEG frequency bands has been shown to be highly correlated with different brain activities. Through that, they found that temporal specific transformations (cutout and delay) are the most effective ones. Last, they investigated two scenarios: (1) using subject-specific distributions to compute the contrastive loss and (2) promoting subject invariance through adversarial training, finding that promoting subject invariance increases classification performance when training with a small number of subjects.

### Data-efficient cardiac arrhythmia classification

Electrocardiogram (ECG) data are ubiquitous in healthcare settings and are increasingly common in personal devices such as the Apple Watch. Kiyasseh et al.^14^ proposed CLOCS, which leverages temporal and spatial invariances of ECG leads based on the two key observations: adjacent ECG segments of shorter duration will continue to share context, and recordings from different leads (at the same time) will reflect the same cardiac function and, thus, share context. A new idea was to define a positive pair as a representation of transformed instances that belong to the same patient. By doing so, the model implicitly personalizes the learned representations to each patient. Driven by this, they designed a new contrastive objective that outperformed supervised and generic self-supervised methods (in terms of AUC) such as BYOL, most notably, with only 25% of labelled training data.

### Improving patient monitoring

Yèche et al. took the idea of inducing priors on contrastive losses a step forward.^21^ They design an objective that preserves the time dependency of the representations of the timeseries segments and outperforms unsupervised and supervised methods in predicting intensive care unit (ICU) decompensation, length of stay, and sepsis onset (on the MIMIC dataset). The versatility of this approach is twofold: when fully unsupervised, it is competitive to supervised models, and when used in a supervised manner, it outperforms contrastive methods. Tonekaboni et al. independently arrived to a similar formulation by ensuring that in the encoding space, the distribution of signals from within a neighborhood is distinguishable from the distribution of nonneighboring signals.^22^ Their models surpassed competitors such as the triplet loss and contrastive predictive coding in predicting diverse outcomes, ranging from atrial fibrillation to human activity recognition, assessed with clustering metrics such as the silhouette score and classification metrics such as the area under the precision-recall curve. They also showed better clusterability over other contrastive losses. Both studies highlighted the generality of such models, which can be reused in multiple downstream tasks.

## Further implications

### Impact on physician workload

Apart from improving accuracy, self-supervision has the potential to lower physicians' workload. Most healthcare systems are overstretched, staff suffer from burnout, and predictive clinical tools could serve to relieve some pressure, especially in increasingly aged populations. These patients require more investigations—such as imaging or ECGs—hence adding to the overload of clinicians. Thus, employing clinical domain experts to label large datasets whilst working in understaffed departments is an expensive and unrealistic expectation that contributes to the bottleneck for the adoption of ML. Radiologists were the first to acknowledge this vicious cycle, which is likely to propagate to other medical specialties. Therefore, it is of paramount importance to develop efficient models that require minimal—or zero—annotations and physician burden. Self-supervised models and the automation of time-consuming tasks are a feasible solution toward more efficient medicine, potentially enabling more scalable patient screening, preventing delays in time-sensitive diagnoses, clinical management decisions, and hence, improving prognoses.

As a result, investing resources only on supervised models will slow down the adoption of ML in clinical settings and will disproportionally benefit high-income countries. Instead, label-efficient models pre-trained on large populations could generalize to global settings, which is of special relevance now that hospitals resume their elective operations whilst still fighting the COVID-19 pandemic.

### Limitations

Self-supervised models tend to achieve remarkable transfer results with a fraction of labeled data. However, training these models can be computationally prohibiting, since it has been empirically shown that they require more data and more training iterations.^5^ Additionally, SSL is a multi-step process, involving a first step of generating augmentations, then training the pretext model, and last, fine-tuning to the target dataset, which makes the entire pipeline more complicated than simple end-to-end supervised models. Additionally, tracking progress in the area of health signals is not trivial, since there are many different tasks, modalities, baselines, and evaluation metrics. For instance, in some papers, the sign when measuring the gap between SSL and supervised models is positive and, in some, negative, which seems to point to benchmark dependencies.

### Conclusion and future outlook

We demonstrated the potential of models that learn meaningful representations directly from unstructured data and presented some recent results in the area of biomedical signals. The underlying challenge here is to find the best representation for biomedical signals, which range from generic ones, such as in BYOL,^10^ to ones based on medical intuition and physiology.^18^^,^^22^ Given that we have strong statistical priors about the nature of these signals, we are particularly excited by the prospect presented by the latter. Zooming out, Andrew Ng has recently expressed some alarm that there is a considerable gap between proof-of-concept models and actual real-life deployments, due to differences in sensors, protocols, or data collection methods: “In contrast, any human [doctor] can walk down the street to the other hospital and do just fine” (https://www.fastcompany.com/90630654/stanford-ai-experts-healthcare). Sequential transfer learning, as seen, for example, in Step2Heart^18^ or in numerous recent works,^10^ is probably the first step to validating that learned representations can generalize across different tasks while being label-efficient. The next steps should focus on demonstrating how these models can perform equally well in changing environments (e.g., different hospitals, populations, or devices). Some exciting new approaches toward this direction include disentangled autoencoders and meta-learning for domain generalization. In short, access to high-quality labeled datasets is the main bottleneck of transferring ML advances to critical fields such as healthcare, and SSL appears to be a feasible solution.
